# Attenuated expression of HRH4 in colorectal carcinomas: a potential influence on tumor growth and progression

**DOI:** 10.1186/1471-2407-11-195

**Published:** 2011-05-24

**Authors:** Zhengyu Fang, Wantong Yao, Yi Xiong, Jiana Li, Li Liu, Lei Shi, Wei Zhang, Chao Zhang, Liping Nie, Jun Wan

**Affiliations:** 1 Biomedical Research Institute, Shenzhen-PKU-HKUST Medical Center, Guangdong Province, Shenzhen, China; 2 Department of Biochemistry and Molecular Biology, Shanghai Medical College, Fudan University, 138 Yi Xue Yuan Road, Shanghai 200032, China; 3 JNU-HKUST joint lab, Ji-Nan University, Guangdong, China; 4 Section of Biochemistry and Cell Biology, Division of Life Science The Hong Kong University of Science and Technology, Clear Water Bay, Kowloon, Hong Kong; 5 Department of Clinical Laboratory, Shenzhen Hospital, Peking University, Guangdong, China

## Abstract

**Background:**

Earlier studies have reported the production of histamine in colorectal cancers (CRCs). The effect of histamine is largely determined locally by the histamine receptor expression pattern. Recent evidence suggests that the expression level of histamine receptor H4 (HRH4) is abnormal in colorectal cancer tissues. However, the role of HRH4 in CRC progression and its clinical relevance is not well understood. The aim of this study is to evaluate the clinical and molecular phenotypes of colorectal tumors with abnormal HRH4 expression.

**Methods:**

Immunoblotting, real-time PCR, immunofluorescence and immunohistochemistry assays were adopted to examine HRH4 expression in case-matched CRC samples (n = 107) and adjacent normal tissues (ANTs). To assess the functions of HRH4 in CRC cells, we established stable HRH4-transfected colorectal cells and examined cell proliferation, colony formation, cell cycle and apoptosis in these cells.

**Results:**

The protein levels of HRH4 were reduced in most of the human CRC samples regardless of grade or Dukes classification. mRNA levels of HRH4 were also reduced in both early-stage and advanced CRC samples. *In vitro *studies showed that HRH4 over-expression caused growth arrest and induced expression of cell cycle proteins in CRC cells upon exposure to histamine through a cAMP -dependent pathway. Furthermore, HRH4 stimulation promoted the 5-Fu-induced cell apoptosis in HRH4-positive colorectal cells.

**Conclusion:**

The results from the current study supported previous findings of HRH4 abnormalities in CRCs. Expression levels of HRH4 could influence the histamine-mediated growth regulation in CRC cells. These findings suggested a potential role of abnormal HRH4 expression in the progression of CRCs and provided some new clues for the application of HRH4-specific agonist or antagonist in the molecular therapy of CRCs.

## Background

Colorectal cancer is the uncontrolled growth of malignant cells in the colon or rectum. It is currently the third most common cancer in the Chinese population, responsible for about 130,000 deaths per year. The growth of CRC cells is influenced by various factors such as insulin-like growth factors (IGF) [1], endothelial growth factor [2,3] and epidermal growth factor receptor [4-6].

Histamine and histamine receptors, previously identified as critical molecules during inflammation, are also involved in the control of CRC growth [7-10]. Histamine is a ubiquitous chemical messenger that exhibits numerous functions and may act as ans [7,11,12]. Histamine levels in cells and tissues are regulated by the activity of histidine decarboxylase (HDC), the only enzyme responsible for the generation of histamine from L-histidine. Therefore, HDC can serve as a specific marker for the biosynthesis of histamine. It has been shown that the levels of HDC mRNA protein and its enzymatic activity are significantly increased in both experimental and human tumors, including colorectal carcinoma [10,13-15].

The biological function of histamine is mediated through at least four pharmacologically distinct receptors, histamine receptor H1-4 (HRH1-4), which are all members of the G-protein-coupled receptor (GPCR) family. HRH1 and H2 have previously been indicated to be correlated with histamine-mediated tumor growth [8,16]. Recently, accumulated evidence indicates that histamine receptor H4 (HRH4) also plays a role in cell proliferation, both in normal and malignant cells, including hematopoietic progenitor cells [17], breast cancer cells [18] and pancreatic carcinoma cells [19].

H4 receptor is positively expressed along the human gastrointestinal tract [20]. Nonetheless, whether HRH4 plays a role in the epithelium of alimental canal or colorectal tumor progression remains unclear. Boer et al. reported the down-regulation of HRH4 expression in human colorectal tumors, which indicated the disturbance of local tumor growth regulation by histamine [21]. However, an earlier study showed a different result when the H4R expression in CRC tissue and the corresponding normal colon mucosa is compared [22]. Therefore, more data from clinical samples are required to establish the role of H4R expression in CRC carcinogenesis.

In the current study, a relatively high number (n = 107) of CRC samples together with matched adjacent normal tissues (ANTs) were collected and used for the examination of H4R expression. We found that both the protein and mRNA levels of HRH4 were decreased in CRC tissues compared with matched ANTs. *In vitro *studies using colorectal cell line showed that alteration of HRH4 expression on colorectal cancer cells affected histamine-mediated cell growth control, implicating the cAMP/PKA pathway in this progress. These findings suggested a potential role of HRH4 abnormalities in CRC progression.

## Methods

### Patients and Tissue Collection

CRC samples were obtained from 107 surgical patients from the Department of Gastroenterology, Shenzhen Hospital, Peking University. Adjacent normal mucosa samples located at least 2 cm from the macroscopically unaffected margins of the tumor (polyp or carcinoma) were defined as normal controls. All tumors that were adenocarcinomas and mucinous carcinomas (when >50% of the tumor volume was composed of mucin) were excluded. Adenocarcinomas were staged according to the Dukes classification system: Dukes A (T_1_-T_2_, N0, and M_0_; n = 21), Dukes B (T_3_-T_4_, N0, and M_0_; n = 36), Dukes C (any T, N_1-2_, M_0_; n = 39) and Dukes D (any T and any N and M_1_; n = 11). Tissue samples for Western blot analysis were placed immediately in the lysis buffer and frozen at -20°C. All patients were informed about the aims of specimen collection and given signed written consent in accordance with the ethical guidelines of Peking University. The study was approved by the ethical committee of Peking University Shenzhen Hospital.

### Cells and culture conditions

The human colorectal cancer cell lines, Colo-320 and Lovo, were obtained from the Department of Biochemistry, Hong Kong University of Science and Technology. The cell lines, whose characteristics are described in detail elsewhere [23,24], were propagated in Dulbecco's modified Eagle's medium (DMEM, Gibco), supplemented with 10% fetal bovine serum (PAA) and 1% penicillin/streptomycin (Life Technologies, Inc.).

### Plasmids and transfection

The cloned HRH4 cDNA fragment was inserted into pcDNA3.1 expression vector to construct the HRH4 expression vector pcDNA3-HRH4. To produce stable transfectants, pcDNA-HRH4 and mock plasmids were stably transfected into the Lovo line using Lipofectamine 2000 reagent (LF2000, Invitrogen, Carlsbad, CA) according to the manufacturer's recommendations. Selection was performed via the addition of 1mg/ml G418. Positive (pcDNA3-HRH4) and negative (empty vector) clones were selected and transfectants with moderate expression levels of HRH4 were used for the experiments described herein. The transfectants from the backbone vector and pcDNA3-HRH4 were designated as mock-Lovo and H4R-Lovo, respectively.

### Western Blotting

Cells were washed with PBS and lysed in a buffer containing 50 mM Tris-HCl (pH 6.8), 1% SDS, 10% glycerol, phosphatase inhibitors (100mM Na_3_VO_4_, 10 mM NaF), and protease inhibitor (1mM PMSF). Equal amounts of protein were loaded onto a SDS-PAGE and transferred to PVDF membrane. After blocking with 5% non-fat milk in TBS-T (containing 0.1% Tween-20), the membranes were incubated with specific primary antibodies, followed by HRP-conjugated secondary antibodies. Proteins were visualized by fluorography using an enhanced chemiluminescence system.

### RT-PCR and Real-time quantitative PCR

Total RNA was isolated using the Trizol system according to the manufacturer's guidelines. Oligo(dT) 18 primer and M-MLV reverse transcriptase were used for first strand synthesis. The cDNA was then used as template for real-time PCR and RT-PCR with gene specific primers. Real-time PCR was performed with Real-time PCR Master Mix containing SYBR GREEN I and hotstart Taq DNApolymerase. GAPDH was amplified as control. The primers for HRH4 and GAPDH are: HRH4 (sense): 5'-GCC TGG GTG TCA ATA AT-3', HRH4 (antisense): '-AGG CAG AGG TTG CAG TGA-', PCR product length was 123 bp; hGAPDH(Sense): 5'-CAG CCT CAA GAT CAT CAG CA-3'; hGAPDH(anti-sense): 5'-TGT GGT CAT GAG TCC TTC CA-3' PCR product length was 105 bp. Real-time detection of the emission intensity of SYBR GREEN bound to double-stranded DNAs was performed using the Icycler Instrument (Bio-rad). At the end point of PCR cycles, melt curves were made to check product purity. The level of ITGA5 mRNA was expressed as a ratio relative to the GAPDH mRNA in each sample.

To determine the amplification efficiency of HRH4 cDNA in this study, a standard curve was prepared using 2 μl of sample cDNA solutions, in which serially diluted samples (original, 2-, 4-, 8-, 16-diluted) were included. The slopes of Ct and dCt ((target gene)-(reference gene)) and R^2 ^values of each sample were calculated by the BioRad Chromo4 real-time PCR system and Microsoft Excel 2007 for Windows. Relative quantification of HRH4 was performed with the 2^-ddCt ^method [25]. The results were obtained from 3 reactions in each sample and analyzed by the Boxplot software.

### Immunofluorescence Staining

We selected a number of CRC samples with significantly attenuated HRH4 expression (according to results from Western blot, n = 15) to make paraffin-embedded tissue sections (4 μm thick). After fixation by 2% paraformaldehyde for 15 min, the coverslips were blocked by rabbit serum (Sigma) for 1 h, followed by incubation with anti-HRH4- antibody (1:50, CHEMICON, US) for 1 h. After washing with 0.01% saponin in PBS 3 times for 15 min each, the coverslips were incubated with secondary antibody conjugated with CY3 (Jackson Immuno Research, US) for another hour. The slips were then washed and DAPI solution was used for nuclear stain. The coverslips were further washed with 0.01% saponin in PBS, 3 times, 15 min each, and PBS twice, 10 min each. Then the coverslips were mounted in an appropriate anti-fade mounting medium. Fluorescence was observed using an Olympus biological fluorescence microscope (IX2-ILL100).

### Immunohistochemistry

Immunohistochemical analysis was performed on 4% formaldehyde-fixed and paraffin-embedded sections of selected samples (n = 20). The paraffin sections were deparaffinized in xylene and hydrated with ethanol. The slides were treated with 3% H_2_O_2 _in methanol for 10 min and blocked with serum blocking solution for 10 min to reduce nonspecific background. The affinity-purified anti-HRH4 rabbit polyclonal antibody (CHEMICON, US, 1:50) was applied to the slides and incubated at room temperature for 1 h. The slides were then incubated with biotinylated goat anti-rabbit secondary antibody (kit supply) at room temperature for 10 min followed by streptavidin peroxidase conjugate for 10 min. 3,3'-Diaminobenzidine (DAB) solution was added as a peroxidase substrate and incubated for 15 min. Cell nuclei were counterstained by hematoxylin to give a blue background contrast to the red color of the positive reaction. The sections were cover-slipped and viewed under fluorescence microscope (Nikon ECLIPSE 80i, JPN).

### Cell Proliferation and cloning-forming (clone-forming?)ability

Cell proliferation was measured by WST-1 assay. Mock-Lovo and H4R-Lovo cells were plated in 96-well culture plates (1×10^4 ^per well) and treated with or without clozapine/histamine. WST-1 (Roche) assay measuring the activity of mitochondrial dehydrogenases was performed following the manufacturer's instruction at 0-, 1-, 2-, 3-, 4-, 5- day time points.

To determine long-term effects, cologenic (clonogenic?) assay was used to elucidate the possible differences in the long-term effects of altered HRH4 expression on human colorectal cancer cells. Mock-Lovo and H4R-Lovo cells were trypsinized and counted using a hemocytometer. Cells (2×10^4^) were plated in the 6-well dishes and supplemented with histamine or clozapine 24 h later. Two weeks after the onset of drug selection, the cells were fixed and stained with crystal violet (0.1% crystal violet in 20% methanol). A cluster of a minimum of 50 cells is considered a colon (clone? colony). (I am not familiar with cancer so please check)

### Flow cytometric analysis of cell cycle

For cell-cycle assay, cells were trypsinized with 2mM EDTA in PBS and rinsed twice with ice-cold PBS solution, then fixed by adding them drop-wise into 75% ice-cold ethanol while vortexing, followed by incubation in ice for 60 min. The fixed cells were washed with ice-cold PBS and incubated at 37°C for 30 min in 0.5 ml PBS solution containing 20 μg/ml RNaseA, 0.2% Triton X-100, 0.2mM EDTA and 20 μg/ml of propidium iodide. The percentage of cells in G0/G1, S, and G2/M phases was determined using the EPICS-XL flow cytometer (Beckman-Coulter, USA) and the Multicycler program.

### Analysis of cell death

Cells were seeded in a growth medium at 1×10^5 ^cells/35-mm dish, allowed to attach overnight, and treated with 10mM 5-Fu for 36 h. Cells were harvested with trypsin, washed once, and resuspended in Dulbecco's modified Eagle's medium. To detect annexin V, cells were incubated with annexin V-EGFP (Genscript, US) for 20 min at 37°C and then washed to remove unbound annexin. Propidium iodide was added, and the cells were incubated for an additional 15 min. The labeled cells were analyzed by flow cytometry. Cells that showed EGFP staining were designated as apoptotic, whereas the double stained cells were designated as post-apoptotic.

TUNEL labeling was also used to examine cell death in cultures exposed to 5-Fu. Cells on coverslips were fixed in 4% paraformaldehyde, pH 7.4, for 30 min and permeabilized for 30 min in 0.1% Triton X-100/PBS at room temperature, and apoptotic nuclei were detected using a TUNEL-labeling reaction according to the manufacturer's instructions (Roche Biochemicals), as described previously[26].

### Statistical analysis

Statistical analysis was performed with the SPSS Software, version 12. Data were analyzed by the chi-square test or Fisher exact test. P values less than 0.05 were considered statistically significant. Results of HRH4 mRNA expression for normal and tumor tissue samples were compared using two-way repeated measurement ANOVA.

## Results

### Impaired HRH4 expression in CRCs in a Chinese population

**Figure 1:**
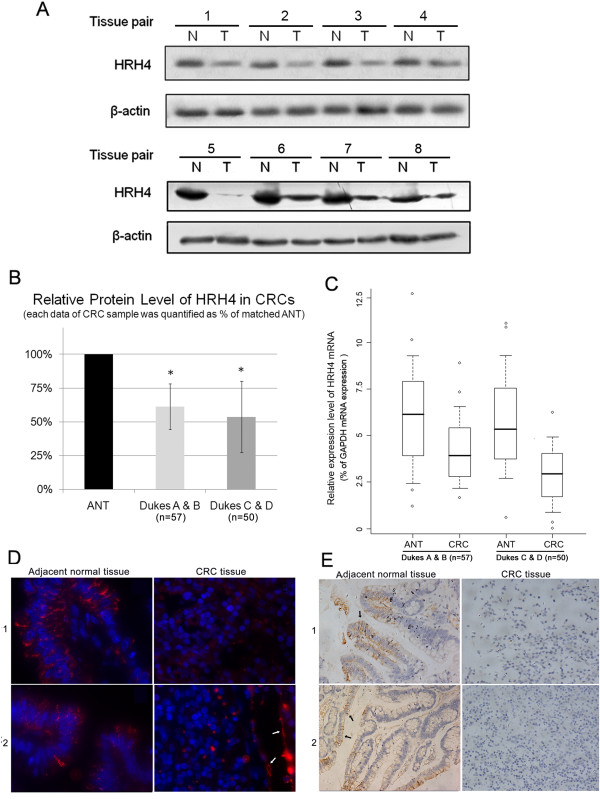
**Attenuated HRH4 expression in CRCs**. (A) Representative blots of HRH4 expression in normal mucosa and colorectal tumor tissues. Sample 1, 2: Dukes A; sample3, 4: Dukes B; sample5, 6: Dukes C; sample7, 8: Dukes D. GAPDH was used as a stable endogenous control. (B) Analysis of HRH4 protein level in CRC samples. Quantitative image of blot were calculated using TotalLab TL100 software, and quantities of GAPDH were used to normalize the HRH4 expression data. The expression level of HRH4 in each sample was calculated as the ratio between CRC tissues and matched adjacent normal tissue. Each data was obtained from two independent results of immunoblottings. (C) Real-time PCR assay was carried out as described under Materials and Methods section, boxplots of relative HRH4 mRNA(HRH4/GAPDH) measured with real-time PCR analysis showing median; box: 25th -75th percentile; bars: largest and smallest values within 1.5 box lengths; little circles: outliers. The results were obtained from 3 reactions in each sample. (D) Representative immunofluorescent microscope analysis of paired samples of CRC tissue and adjacent normal tissue using anti human HRH4 monoclonal antibody (red). Nuclei were stain with DAPI (blue). Sample1: rectum tissue; sample2: colon tissue, white arrow points to the positive control of HRH4 expression on blood vessel epithelium. (E) Representative immunohistochemical staining of paired samples of CRC tissue and adjacent normal tissue using anti human HRH4 monoclonal antibody. Sample1: rectum; sample2: colon tissue. Black arrow points to the positive expression of HRH4 expression on enterocytes.

We first examined the H4R protein levels in the case-matched CRC samples and adjacent normal tissues. As shown in Figure 1A &1B, attenuated expression levels of HRH4 were detected in most CRC samples compared to matched ANTs, regardless of the Dukes classification. Based on these results, we performed real-time RT-PCR, immunocytochemistry, and immunohistochemical analysis of the matched adjacent normal mucosa and adenocarcinoma samples. It was found that HRH4 mRNA levels were also significantly reduced in the CRCs (Figure 1C, p < 0.001), and there was a statistical difference between the group of early-stage CRCs and advanced CRCs (p < 0.05). Some of the results were confirmed by RT-PCR, which were shown in Additional file 1 Figure 1. Figure 1D &1E showed the representative immunocytochemistry and immunohistochemical analysis of HRH4 expression in selected CRC samples. The results obtained were in accordance with the data from the immunoblotting.

### Restoration of HRH4 in colorectal cancer cell line resulted in impaired proliferation upon exposure to HRH4 agonist

**Figure 2:**
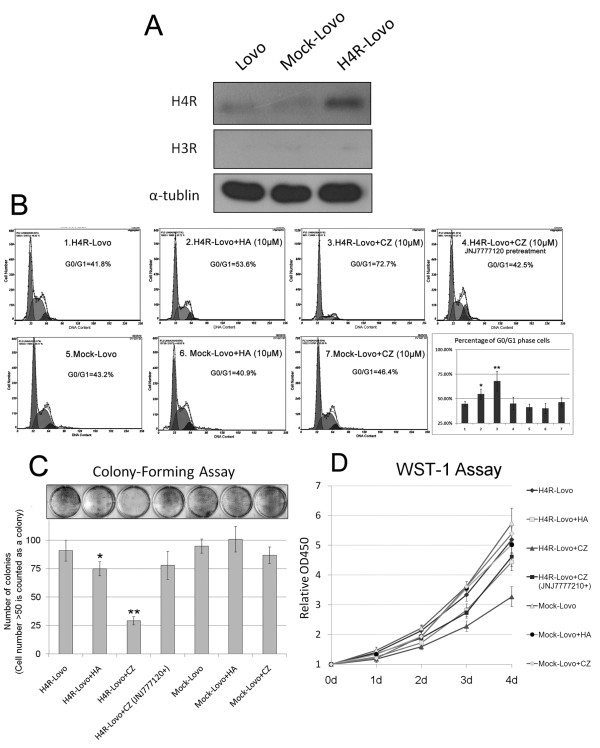
**HRH4 activation induced growth arrest in colorectal carcinoma cell lines**. (A) Total cell lysates of wild type Lovo, Mock-Lovo and H4R-Lovo cells were examined by Western blotting. (B) Mock-Lovo and H4R-Lovo cells were treated with 10^-5^M histamine, clozapine or clozapine accompanied by HRH4 antagonist pretreatment, and cell-cycle distributions were determined by propidium iodide flow cytometry analysis. *p < 0.05 and **p < 0.01 vs. Control, H4R-Lovo cells without any treatment. (C) Colony-formation assay. 5×10^3 ^Mock-Lovo and H4R-Lovo cells were treated as in Fig.2B and maintained in G418 for 14 days, and the colonies were stained with Giemsa. The bar graph shows the absolute colony (≥50 cells) number ± s.e. in duplicate experiments. (D) H4R-Lovo cells were treated as in Fig.2B, WST-1 (Roche) assay measuring the activity of mitochondrial dehydrogenases was performed following the manufacturer's instruction at 0-, 1-, 2-, 3-, 4-, 5- day time points. Error bars represent standard deviation of the mean.

To investigate the role of impaired HRH4 expression in colorectal cancer, *in vitro *experiments using CRC cell lines were performed. We first assessed the expression of HRH4 in 12 colorectal cancer cell lines (Additional file 1 Figure S2). The cell line with relatively lower expression of HRH4, Lovo line, was stably transfected with HRH4 expression construct and then named as H4R-Lovo. Lovo cells transfected with the backbone vector were named as mock-Lovo (Figure 2A).

HRH4 has been implicated in cell growth control in various types of cells [17-19]. Here we evaluated whether manipulation of the activation level of HRH4 regulates cell cycle status of the stable cell lines. We used histamine, the natural ligand of HRH4, and clozapine, one of the most specific agonist of HRH4, to activate HRH4. Although clozapine was capable of activating both HRH3 and H4, it could be used as HRH4-specific agonist here because there was no appreciable H3R expression in Mock-Lovo or H4R-Lovo cells (Figure 2A). It was found that both histamine and clozapine inhibited proliferation of H4R-Lovo cells at an optimal dose of 10^-5 ^M, as illustrated by the accumulation of cells at stage G0/G1 and a decrease at stage G2/S, when either histamine or clozapine treatment has little influence on Mock-Lovo cells (Figure 2B) (This sentence is a little hard to understand. Need to rephrase). Moreover, pre-treatment with the selective HRH4 antagonist JNJ7777120 before exposure to histamine or clozapine prevented the cell cycle arrest, providing additional evidence that activation of HRH4 leads to cell cycle arrest.

To determine whether the alteration of HRH4 expression in Lovo cells affects long-term tumor growth, we evaluated the growth rate of H4R-Lovo and mock-Lovo cells that were incubated with the medium containing histamine (10^-5^M) or clozapine (10^-5^M). As shown in Figure 2C, cells transfected with the HRH4 gene generated a lower number of colonies than those transfected with the backbone vector after HRH4 agonist treatment. Moreover, we analyzed the growth potential of H4R-Lovo cells by WST-1 assay at different times after plating. It was found that H4R-Lovo cells grew at a significantly slower rate when incubated with a medium containing histamine or clozapine, while growth of Mock-Lovo cells was not influenced by the HRH4 agonist (Figure 2D).

We also used another specific HRH4 agonist, clobenpropit (CB), to investigate the role of HRH4 in colon cancer proliferation, and similar results were obtained (A part of results were shown in Additional file 1 Figure S3).

### Effect of HRH4 activation on cell-cycle regulatory molecules

**Figure 3:**
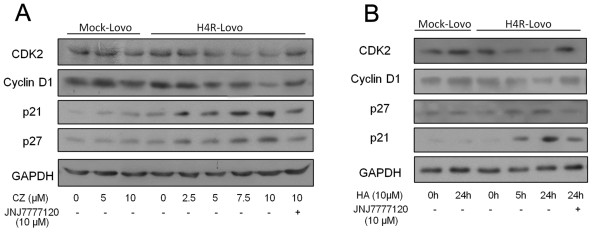
**Expression of cell cycle proteins regulated by HRH4**. (A) Mock-Lovo and H4R-Lovo were treated with clozapine at different doses for 24h, and total cell lysates were examined by western blotting. One dish of H4R-lovo cells was pretreated with 10^-5^M JNJ7777120 for 2 h. (B) Mock-Lovo and H4R-Lovo were treated with 10^-5^M histamine at for 5 h or 24 h, and total cell lysates were examined by western blotting.

The clozapine-induced G1 arrest was further confirmed by examining the cellular levels of the G1 cell-cycle control proteins cyclin D1 and Cdk2 in H4R-Lovo cells. Western immunoblotting analysis confirmed that treatment with 10^-5^M clozapine down-regulated the levels of cyclin D1 and Cdk2 proteins in H4R-Lovo cells and the expression of p21^Cip1 ^and p27^Kip1 ^was substantially up-regulated following 24h of clozapine stimulation in a dose-dependent manner (Figure 3A). Based on these findings, clozapine-mediated activation of HRH4 likely blocks cell-cycle progression from the G1 to S phase. Relatively high concentrations of histamine (Figure 3B) mimics the effect of clozapine, suggesting the potential role of increased histamine production and reduced HRH4 expression in the tumor growth of CRCs. Expression levels of cycle proteins in Mock-H4R cells were not significantly influenced by clozapine or histamine treatment, which further confirmed the role of HRH4 in this process.

### HRH4-mediated cell cycle control is mediated through cAMP/PKA-dependent signaling

Activation of endogenously expressed H4 receptors in eosinophils and mast cells leads to PTX-sensitive calcium mobilization [27,28], via the activation of phospholipase C. In human hematopoietic progenitor cells, T cell or some cell lines recombinantly express H4 receptor. However, HRH4 is coupled with pertussis toxin (PTX)-sensitive Gαi/o proteins, therefore inhibiting forskolin-induced cAMP and its-responsive elements[17,29].

**Figure 4:**
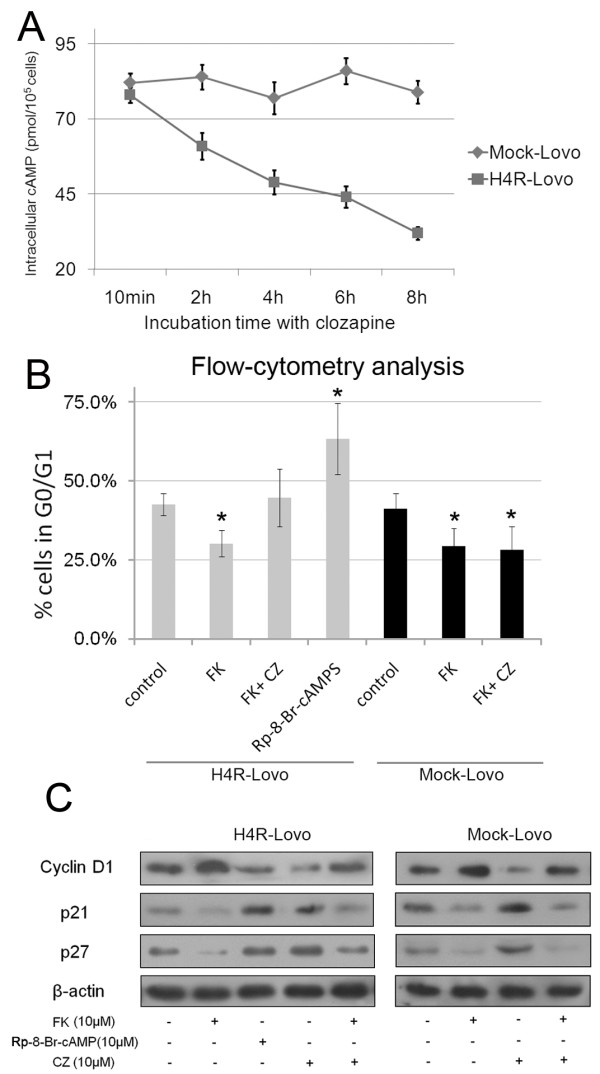
**HRH4 activation regulates proliferation of colon cancer cell through a cAMP-dependent pathway**. (A) Mock-Lovo and H4R-Lovo cells were exposed to 5mM clozapine for 10 min to 8 h. Intracellular cAMP levels were determined by cAMP Enzyme Immunoassay Kit as introduced in Material and Methods. Values are mean ± S.D. of two experiments performed in triplicate. (B) H4R-Lovo and Mock-Lovo cells were treated with 10-5M forskolin, forskolin together with clozapine or Rp-8-Br-cAMPS, and cell-cycle distributions were determined by propidium iodide flow cytometry analysis. *p < 0.05 vs. Control, cells without any treatment. (C) H4R-Lovo and Mock cells were treated with clozapine, clozapine together with FK or Rp-8-Br-cAMPS as in Fig.4B, and total cell lysates were examined by Western blotting.

To elucidate the molecular basis for clozapine-induced cell cycle arrest, we first examined whether cAMP/PKA pathway was involved in HRH4-mediated cell cycle control. As shown in Figure 4A, activation of HRH4 suppressed the level of intracellular cAMP in H4R-Lovo cells. Clozapine inhibited cell cycle progression induced by forskolin, a chemical commonly used to raise intracellular cAMP levels (Figure 4B). Also, the cAMP inhibitor Rp-8-Br-cAMPS mimicked the cell cycle arrest induced by CZ in H4R-Lovo cells. Clozpine could not block the effect of forskolin in Mock-Lovo cells, which further indicated the specific role of HRH4 in the regulation of cAMP pathway.

We also examined the expression levels of cell cycle proteins in H4R-Lovo and Mock-Lovo cells treated with cAMP inhibitors and agonists (Figure 4C). The results were in accordance with those from flow cytometry analysis. Clozapine blocked forskolin-induced cell cycle progression in H4R-Lovo cells but not in Mock-Lovo cells. This further supported the involvement of the cAMP/PKA pathway in HRH4-mediated cell cycle regulation.

### HRH4 activation could promote apoptosis induced by 5-Fu treatment in colon cancer cells

**Figure 5:**
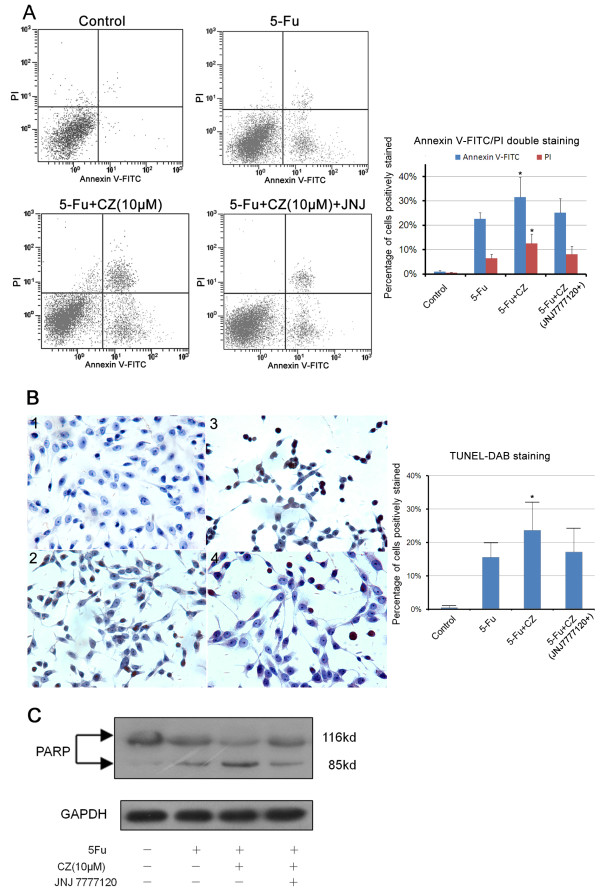
**HRH4 activation promotes 5-Fu-induced cell apoptosis in colon cancer cells**. (A) H4R-Lovo cells were treated with 10mM 5-Fu, 5-Fu together with clozapine(10^-5^M) or 5-Fu (10mM) and clozapine with JNJ7777120(10^-5^M) pretreatment, and cell apoptosis was assessed using annexin V/PI staining and flow cytometry. *p < 0.05 vs. control, cells treated with 5-Fu (10mM) alone. (B) H4R-Lovo cells were treated as in Fig.5A, and TUNEL-DAB staining was performed according to the guidance of the kit. *p < 0.05 vs. control, cells treated with 5-Fu alone. (C) H4R-Lovo cells were treated as in Fig.5A, and total cell lysates were examined by Western blotting.

HRH4 expression has been implicated in cell apoptosis [30], and HRH4 agonist has been reported to induce early-stage apoptosis in peripheral blood mononuclear cells (PBMCs) [29]. Thus cell apoptosis may also be involved in the H4R-mediated regulation of CRC growth. In our preliminary work, the treatment of clozapine or histamine could not directly induce evident apoptosis in H4R-Lovo or Mock-Lovo cells. As an initial approach to identify the influence of cell death by HRH4 activity in CRCs, we treated H4R-Lovo cells with 5-fluorouracil (5-Fu, an anti-cancer drug proved to be an inducer of both early and late stages of apoptosis in colorectal cancer cells including Lovo line [31,32]) to induce cell death. As shown in Figure 5A, incubation of H4R-Lovo cells with clozapine and 5-Fu increased both early and late stage of apoptosis, which was reversed by HRH4 antagonist pre-treatment. We also used TUNEL assay as another method to evaluate apoptosis. Staining of H4R-Lovo cells in the presence of clozapine following 5-Fu treatment revealed augmented TUNEL-positive cells (Figure 5B), which could be blocked by pre-treatment with JNJ7777120. In addition, treatment with 5-Fu together with clozapine resulted in enhanced levels of cleaved (89 kDa) PARP in H4R-Lovo cells (Figure 5C). These findings suggested a potential role of HRH4 in the apoptosis of CRC cells.

## Discussion

The distribution of HRH1, HRH2 and HRH4 in human intestinal tract has been described [20]. While the role of H1R and H2R in different gastrointestinal tumor models is well documented [11], the relevance of HRH4 has yet to be clarified. Moreover, only limited data on the level of HRH4 expression in colorectal tumors have been reported. In this study, a relatively large number of CRC samples with different Dukes classifications were used for HRH4 analysis. Attenuated H4R expression was observed in most CRC samples compared with case-matched ANTs, which supported a previous report [21]. The mRNA levels of HRH4 were also examined, which provided additional evidence of transcriptionally repressed HRH4 gene in CRCs. Interestingly, we found that expression levels of HRH4 mRNA were lower in advanced CRCs (p < 0.05), which suggested that decrease of H4R expression might mainly take place during CRC progression but not during initiation. The immunohistochemical and immunoflourescent approach further confirmed the results from immunoblotting and provided additional evidence for the positive expression of H4R on normal enterocytes. These details may help us answer the question of whether direct or indirect effects of histamine, or both, mediated by HRH4, are responsible for tumor progression in colorectal carcinoma.

To the best of our knowledge, no functional elucidation of HRH4 expression in colorectal enterocytes has been described. In the current study, results from the *in vitro *experiments using CRC cell lines first indicated the influence of HRH4 abnormalities on histamine-mediated regulation of CRC growth. Another CRC cell line with low endogenous HRH4 expression, CACO-2, was also transfected with H4R expression vector and used for cell cycle analysis. Similar results were obtained (Additonal file1, Figure S4), which further supported the role of HRH4 expression in growth control of CRC cells.

HRH4 stimulation resulted in cell growth arrest and increase of cyclin-dependent kinase inhibitor p21^Cip1 ^and p27 ^Kip1^, cell cycle regulators with important functions in cell cycle control and apoptosis of colorectal cancer cells [33]. p21^Cip1 ^and p27^Kip1 ^play important roles in mediating growth arrest and are considered to function as brakes of the cell cycle [34]. Extensive studies have been published on the roles of p21^Cip1 ^and p27^Kip1 ^in carcinogenesis of tumors, including colorectal cancer [33,35,36]. The increase in p21^Cip1 ^and p27 ^Kip1 ^could, by inhibiting cyclin D1/Cdk4 or Cdk6 kinase activity, explain, at least partially, the increase of cells in the G1 phase following clozapine treatment in our study. Expression of p21 and p27 in H4R-Lovo cells were also modulated by the use of a cAMP inhibitor. This implies that a link exists between cAMP suppression and p21 and p27 induction by HRH4 activation. However, the inhibitor of PKA had little effect on the expression of p21 and p27 (data not shown here), indicating that cAMP-mediated regulation of CKIs expression was independent of PKA activity. This was consistent with a previous report [37].

HRH4 has been reported to influence apoptosis in animal models of sepsis through counteracting the anti-apoptotic action of NF-kappaB [30], while the cAMP-PKA pathway is involved in HRH4 activation-mediated cell death in peripheral blood mononuclear cells (PBMCs) [29]. In the current study, we showed that HRH4 activation could promote the 5-Fu-mediated cell apoptosis in colon cancer cells, which may provide new clues for histamine receptor-targeted therapies of CRCs. However, the molecular mechanisms involved in the regulation of CRC cell apoptosis by HRH4 require further investigation.

Overall, our results confirmed the down-regulation of HRH4 expression in colorectal malignancies and suggested a potential role of histamine-mediated growth control in CRC cells. We also showed that expression levels of HRH4 in CRC cells had an influence on cell apoptosis induced by chemotherapeutic agent (s?). It will be interesting to perform *in vivo *study aimed at understanding the mechanisms of the HRH4 expression levels using HRH4 KO mice in the future.

## Conclusion

The results from the current study supported previous findings of HRH4 abnormalities in CRCs. Expression levels of HRH4 could influence the histamine-mediated growth regulation in CRC cells. These findings suggested a potential role of abnormal HRH4 expression in the progression of CRCs and provided some new clues for the application of HRH4-specific agonist or antagonist in the molecular therapy of CRCs.

## Competing interests

We declare that we have no financial and personal relationships with other people or organizations that can inappropriately influence our work. There are no professional or other personal interest of any nature or kind in any product, service and/or company that could be construed as influencing the position presented in, or the review of, the manuscript entitled, "Attenuated expression of HRH4 in colorectal carcinomas: a potential influence on tumor growth and progression".

## Authors' contributions

FZY examined the expression levels of H4R in CRC samples and carried out the analysis of cell death, participated in the cell proliferation assays and signaling pathway analysis, and drafted the manuscript. YWT and XY carried out the immunofluorescence staining as well as immunohistochemistry and participated in statistical analysis. LJN, LL, and ZC collected clinical CRC samples, carried out Western blot analysis, and participated in cell culture and transfection. ZW carried out the real-time PCR analysis and participated in statistical analysis. SL participated in signaling pathway analysis and helped to draft the manuscript. NLP carried out the flow cytometry analysis. WJ conceived the study, participated in its design and coordination, and helped to draft the manuscript. All authors read and approved the final manuscript.

## Pre-publication history

The pre-publication history for this paper can be accessed here:

http://www.biomedcentral.com/1471-2407/11/195/prepub

## Supplementary Material

Additional file 1 **Supplementary figures**. Figure S1: Comparison of HRH4 mRNA expression between matched CRC tissues and adjacent normal tissues using RT-PCR assay. GAPDH is used as the internal control. Shown is representative example of multiple experiments; Figure S2: mRNA levels of HRH4 in the colorectal cell lines were analyzed using RT-PCR assay, and normalized with the amount of GAPDH. Shown is representative example of multiple experiments. Figure S3: H4R-Lovo cells were treated with 10^-5^M HA, CB or CB together with JNJ7777120, and cell-cycle distributions were determined by propidium iodide flow cytometry analysis. *p < 0.05 vs. Control, H4R-Lovo cells without any treatment. **p < 0.001 vs. Control, H4R-Lovo cells without any treatment. Figure S4: HRH4 activation blocked cell cycle progression in CACO-2 cells. CACO-2 cells were transiently transfected with HRH4 expression vector for 24h. The wild-type CACO-2 cells and HRH4 transfectants were both treated with 10^-5^M histamine or clozapine for 24h. Cell-cycle distributions were determined by propidium iodide flow cytometry analysis. *p < 0.05 vs. Control, cells without any treatment; **p < 0.001 vs. Control, cells without any treatment. Click here for file

## References

[B1] Yang X Wan DS [Roles of insulin-like growth factor system in colorectal carcinoma and its applications] Ai Zheng 2005 24 1161 1164 16159447

[B2] Collins TS Hurwitz HI Targeting vascular endothelial growth factor and angiogenesis for the treatment of colorectal cancer Semin Oncol 2005 32 61 68 10.1053/j.seminoncol.2004.09.026 15726507

[B3] Guba M Seeliger H Kleespies A Jauch KW Bruns C Vascular endothelial growth factor in colorectal cancer Int J Colorectal Dis 2004 19 510 517 10.1007/s00384-003-0576-y 14999511

[B4] Venook AP Epidermal growth factor receptor-targeted treatment for advanced colorectal carcinoma Cancer 2005 103 2435 2446 10.1002/cncr.21123 15880563

[B5] Kurtin SE Targeting the epidermal growth factor receptor in colorectal carcinoma Cancer Nurs 2007 30 S1 9 10.1097/01.NCC.0000281757.78081.74 17666985

[B6] Yarom N Jonker DJ The role of the epidermal growth factor receptor in the mechanism and treatment of colorectal cancer Discov Med 11 95 105 21356164

[B7] Medina MA Quesada AR Nunez de Castro I Sanchez-Jimenez F Histamine, polyamines, and cancer Biochem Pharmacol 1999 57 1341 1344 10.1016/s0006-2952(99)00005-2 10353253

[B8] Suonio E Tuomisto L Alhava E Effects of histamine, H1, H2 and Hic receptor antagonists and alpha-fluoromethylhistidine on the growth of human colorectal cancer in the subrenal capsule assay Agents Actions 1994 41 Spec No C118 120 10.1007/BF02007793 7976795

[B9] Masini E Fabbroni V Giannini L Vannacci A Messerini L Perna F Cortesini C Cianchi F Histamine and histidine decarboxylase up-regulation in colorectal cancer: correlation with tumor stage Inflamm Res 2005 54 Suppl 1 S80 81 10.1007/s00011-004-0437-3 15928846

[B10] Hellstrand K Brune M Mellqvist UH Naredi P Histamine, cimetidine and colorectal cancer Nat Med 1996 2 364 365 10.1038/nm0496-364a 8597928

[B11] Molnar EL Cricco G Martin G Darvas Z Hegyesi H Fitzsimons C Bergoc R Falus A Rivera E Histamine as a potential autocrine regulator of melanoma Inflamm Res 2001 50 Suppl 2 S102 103 10.1007/PL00020786 11411574

[B12] Malaviya R Uckun FM Histamine as an autocrine regulator of leukemic cell proliferation Leuk Lymphoma 2000 36 367 373 10.3109/10428190009148858 10674909

[B13] Garcia-Caballero M Neugebauer E Campos R Nunez de Castro I Vara-Thorbeck C Increased histidine decarboxylase (HDC) activity in human colorectal cancer: results of a study on ten patients Agents Actions 1988 23 357 360 10.1007/BF02142587 3394588

[B14] Reynolds JL Akhter J Adams WJ Morris DL Histamine content in colorectal cancer. Are there sufficient levels of histamine to affect lymphocyte function? Eur J Surg Oncol 1997 23 224 227 10.1016/s0748-7983(97)92388-x 9236896

[B15] Backhaus B Weidenhiller M Bijlsma P Hahn EG Raithel M Evaluation of spontaneous histamine release from colorectal mucosa in patients with colorectal adenoma, patients with gastrointestinally mediated allergy and in a healthy control group Inflamm Res 2004 53 Suppl 1 S87 88 10.1007/s00011-003-0342-1 15054633

[B16] Nielsen HJ Hammer JH Moesgaard F Kehlet H Possible role of histamine-2 receptor antagonists for adjuvant treatment in colorectal cancer. Clinical review Eur J Surg 1991 157 437 441 1681927

[B17] Petit-Bertron AF Machavoine F Defresne MP Gillard M Chatelain P Mistry P Schneider E Dy M H4 histamine receptors mediate cell cycle arrest in growth factor-induced murine and human hematopoietic progenitor cells PLoS One 2009 4 e6504 10.1371/journal.pone.0006504 PMC2720606 19662098

[B18] Medina V Croci M Crescenti E Mohamad N Sanchez-Jimenez F Massari N Nunez M Cricco G Martin G Bergoc R Rivera E The role of histamine in human mammary carcinogenesis: H3 and H4 receptors as potential therapeutic targets for breast cancer treatment Cancer Biol Ther 2008 7 28 35 10.4161/cbt.7.1.5123 17932461

[B19] Cricco GP Mohamad NA Sambuco LA Genre F Croci M Gutierrez AS Medina VA Bergoc RM Rivera ES Martin GA Histamine regulates pancreatic carcinoma cell growth through H3 and H4 receptors Inflamm Res 2008 57 Suppl 1 S23 24 10.1007/s00011-007-0611-5 18345506

[B20] Sander LE Lorentz A Sellge G Coeffier M Neipp M Veres T Frieling T Meier PN Manns MP Bischoff SC Selective expression of histamine receptors H1R, H2R, and H4R, but not H3R, in the human intestinal tract Gut 2006 55 498 504 10.1136/gut.2004.061762 PMC1856162 16299042

[B21] Boer K Helinger E Helinger A Pocza P Pos Z Demeter P Baranyai Z Dede K Darvas Z Falus A Decreased expression of histamine H1 and H4 receptors suggests disturbance of local regulation in human colorectal tumours by histamine Eur J Cell Biol 2008 87 227 236 10.1016/j.ejcb.2007.12.003 18258331

[B22] Cianchi F Cortesini C Schiavone N Perna F Magnelli L Fanti E Bani D Messerini L Fabbroni V Perigli G The role of cyclooxygenase-2 in mediating the effects of histamine on cell proliferation and vascular endothelial growth factor production in colorectal cancer Clin Cancer Res 2005 11 6807 6815 10.1158/1078-0432.CCR-05-0675 16203768

[B23] Demicheli R Foroni R Ingrosso A Pratesi G Soranzo C Tortoreto M An exponential-Gompertzian description of LoVo cell tumor growth from in vivo and in vitro data Cancer Res 1989 49 6543 6546 2819710

[B24] Quinn LA Moore GE Morgan RT Woods LK Cell lines from human colon carcinoma with unusual cell products, double minutes, and homogeneously staining regions Cancer Res 1979 39 4914 4924 498117

[B25] Livak KJ Schmittgen TD Analysis of relative gene expression data using real-time quantitative PCR and the 2(-Delta Delta C(T)) Method Methods 2001 25 402 408 10.1006/meth.2001.1262 11846609

[B26] Chen Y Liu H Liu Z Liang S Chen J Long F Peng Y Yan L Gong J Blockade of inducible costimulator pathway to prevent acute rejection in rat liver transplantation Am J Surg 2009 198 244 249 10.1016/j.amjsurg.2008.09.014 19628063

[B27] Buckland KF Williams TJ Conroy DM Histamine induces cytoskeletal changes in human eosinophils via the H(4) receptor Br J Pharmacol 2003 140 1117 1127 10.1038/sj.bjp.0705530 PMC1574117 14530216

[B28] Hofstra CL Desai PJ Thurmond RL Fung-Leung WP Histamine H4 receptor mediates chemotaxis and calcium mobilization of mast cells J Pharmacol Exp Ther 2003 305 1212 1221 10.1124/jpet.102.046581 12626656

[B29] Sugata Y Okano M Fujiwara T Matsumoto R Hattori H Yamamoto M Nishibori M Nishizaki K Histamine H4 receptor agonists have more activities than H4 agonism in antigen-specific human T-cell responses Immunology 2007 121 266 275 10.1111/j.1365-2567.2007.02574.x PMC2265937 17346280

[B30] Matsuda N Teramae H Futatsugi M Takano K Yamamoto S Tomita K Suzuki T Yokoo H Koike K Hattori Y Up-regulation of histamine H4 receptors contributes to splenic apoptosis in septic mice: counteraction of the antiapoptotic action of nuclear factor-kappaB J Pharmacol Exp Ther 332 730 737 10.1124/jpet.109.163543 20008488

[B31] Werner JM Eger K Jurgen Steinfelder H Comparison of the rapid pro-apoptotic effect of trans-beta-nitrostyrenes with delayed apoptosis induced by the standard agent 5-fluorouracil in colon cancer cells Apoptosis 2007 12 235 246 10.1007/s10495-006-0530-x 17136318

[B32] Backus HH Pinedo HM Wouters D Kuiper CM Jansen G van Groeningen CJ Peters GJ Differences in the induction of DNA damage, cell cycle arrest, and cell death by 5-fluorouracil and antifolates Oncol Res 2000 12 231 239 10.3727/096504001108747729 11417748

[B33] Cheng JD Werness BA Babb JS Meropol NJ Paradoxical correlations of cyclin-dependent kinase inhibitors p21waf1/cip1 and p27kip1 in metastatic colorectal carcinoma Clin Cancer Res 1999 5 1057 1062 10353738

[B34] Sherr CJ Roberts JM CDK inhibitors: positive and negative regulators of G1-phase progression Genes Dev 1999 13 1501 1512 10.1101/gad.13.12.1501 10385618

[B35] Cam WR Masaki T Shiratori TY Kato N Okamoto M Yamaji Y Igarashi K Sano T Omata M Activation of cyclin E-dependent kinase activity in colorectal cancer Dig Dis Sci 2001 46 2187 2198 10.1023/a:1011962915280 11680595

[B36] McKay JA Douglas JJ Ross VG Curran S Ahmed FY Loane JF Murray GI McLeod HL Expression of cell cycle control proteins in primary colorectal tumors does not always predict expression in lymph node metastases Clin Cancer Res 2000 6 1113 1118 10741741

[B37] Rao S Gray-Bablin J Herliczek TW Keyomarsi K The biphasic induction of p21 and p27 in breast cancer cells by modulators of cAMP is posttranscriptionally regulated and independent of the PKA pathway Exp Cell Res 1999 252 211 223 10.1006/excr.1999.4620 10502413

