# Supplementary material for: Attenuated expression of HRH4 in colorectal carcinomas: a potential influence on tumor growth and progression
Source: BMC Cancer. 2011 May 24;11:195. doi: 10.1186/1471-2407-11-195 (PMC3128004; doi:10.1186/1471-2407-11-195)
Supplement: Additional file 1 — Supplementary figures. Figure S1: Comparison of HRH4 mRNA expression between matched CRC tissues and adjacent normal tissues using RT-PCR assay. GAPDH is used as the internal control. Shown is representative example of multiple experiments; Figure S2: mRNA levels of HRH4 in the colorectal cell lines were analyzed using RT-PCR assay, and normalized with the amount of GAPDH. Shown is representative example of multiple experiments. Figure S3: H4R-Lovo cells were treated with 10-5M HA, CB or CB together with JNJ7777120, and cell-cycle distributions were determined by propidium iodide flow cytometry analysis. *p < 0.05 vs. Control, H4R-Lovo cells without any treatment. **p < 0.001 vs. Control, H4R-Lovo cells without any treatment. Figure S4: HRH4 activation blocked cell cycle progression in CACO-2 cells. CACO-2 cells were transiently transfected with HRH4 expression vector for 24h. The wild-type CACO-2 cells and HRH4 transfectants were both treated with 10-5M histamine or clozapine for 24h. Cell-cycle distributions were determined by propidium iodide flow cytometry analysis. *p < 0.05 vs. Control, cells without any treatment; **p < 0.001 vs. Control, cells without any treatment. [file 1471-2407-11-195-S1.DOC]

Supplementary figures

Figure S1: Detection of HRH4 mRNA levels in CRC samples using RT-PCR.


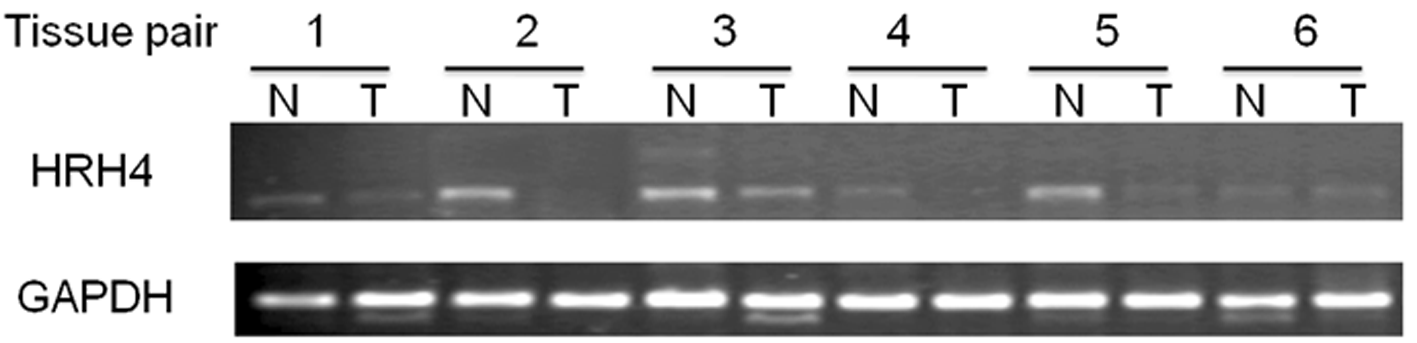


Comparison of HRH4 mRNA expression between matched CRC tissues and adjacent normal tissues using RT-PCR assay. GAPDH is used as the internal control. Shown is representative example of multiple experiments.

Figure S2: Expression levels of HRH4 in different cancer cell lines.


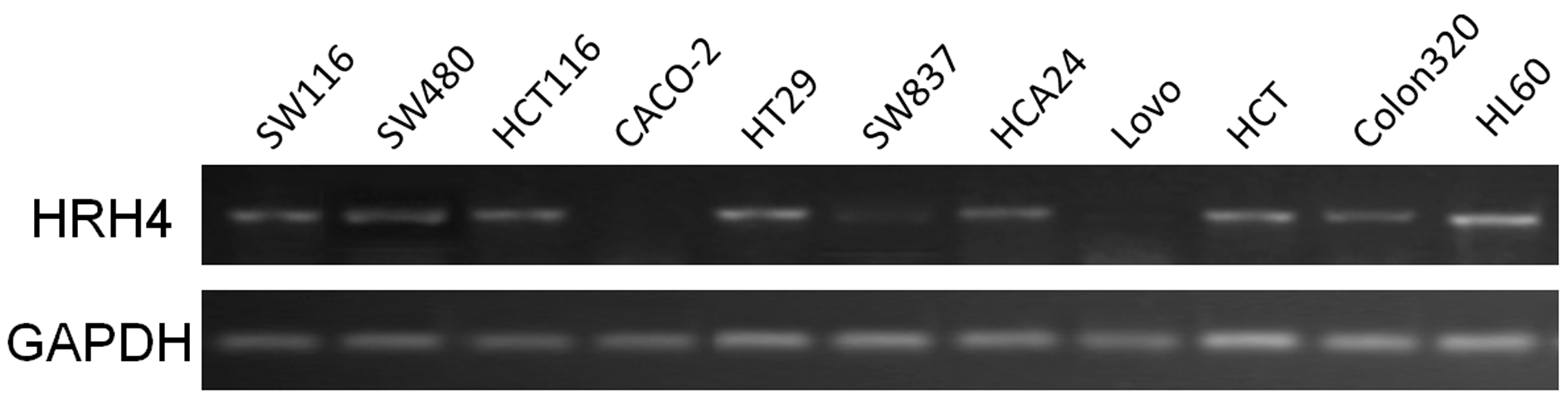


mRNA levels of HRH4 in the colorectal cell lines were analyzed using RT-PCR assay, and normalized with the amount of GAPDH. Shown is representative example of multiple experiments.

Figure S3: Effect of HRH4 activation (using clobenpropit) on Lovo cells.


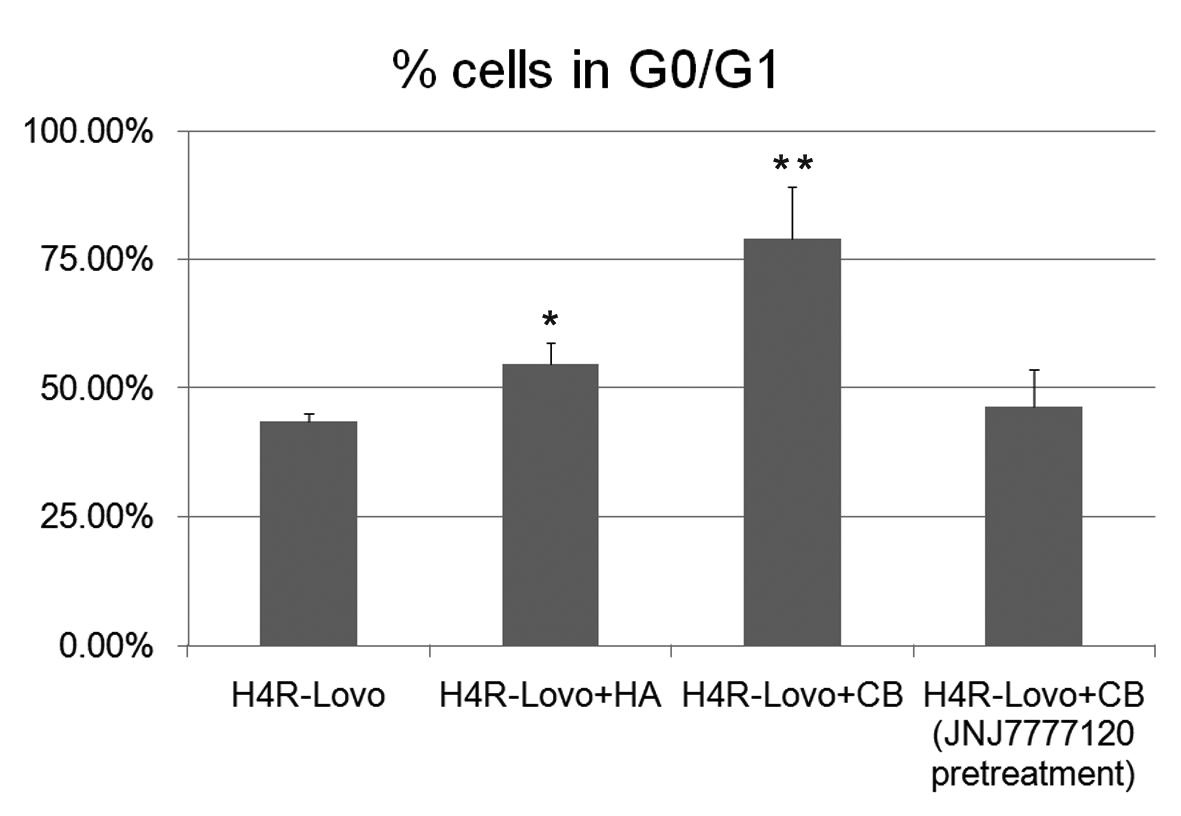


H4R-Lovo cells were treated with 10-5M HA, CB or CB together with JNJ7777120, and cell-cycle distributions were determined by propidium iodide flow cytometry analysis. *p<0.05 vs. Control, H4R-Lovo cells without any treatment. **p<0.001 vs. Control, H4R-Lovo cells without any treatment.

Figure S4: HRH4 activity influences cell cycle in CACO-2 line


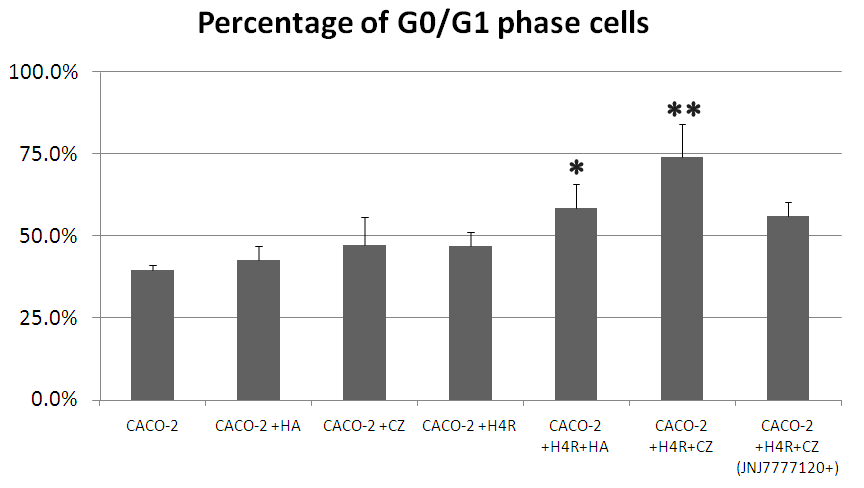


HRH4 activation blocked cell cycle progression in CACO-2 cells. CACO-2 cells were transiently transfected with HRH4 expression vector for 24h. The wild-type CACO-2 cells and HRH4 transfectants were both treated with 10-5M histamine or clozapine for 24h. Cell-cycle distributions were determined by propidium iodide flow cytometry analysis. *p<0.05 vs. Control, cells without any treatment; **p<0.001 vs. Control, cells without any treatment.
